# Video sleds effectively survey epibenthic communities at dredged material disposal sites

**DOI:** 10.1007/s10661-019-7348-9

**Published:** 2019-05-29

**Authors:** Stephanie Fields, Sarah Henkel, G. Curtis Roegner

**Affiliations:** 1 Hatfield Marine Science Center, Oregon State University, 2030 SE Marine Science Drive, Newport, OR 97365 USA; 2 Hatfield Marine Science Center, Oregon State University, 2030 SE Marine Science Drive, Newport, OR 97365 USA; 3 NOAA Fisheries, Point Adams Research Station, 520 Heceta Place, Hammond, OR 97121-0155 USA

**Keywords:** Video sled, Dredged material disposal, Columbia River, Dungeness crab, Epibenthos

## Abstract

This research assessed the effectiveness of benthic video sleds for monitoring the impacts of dredged material disposal on epifauna at shallow and deep water disposal sites near the mouth of the Columbia River, USA. Video sleds collected visual transects at the two disposal sites and comparable reference areas during 2014 and 2015 within a Before-After, Control-Impact (BACI) experimental design. These flat, soft-bottom habitats are populated by demersal fish and benthic invertebrates, including the economically important Dungeness crab (*Cancer magister*). At the shallow site, results from multivariate Similarity Profile Analysis (SIMPROF) and univariate ANOVA tests on prominent species did not detect any significant differences between disposal and reference communities. At the deep site, the multivariate and univariate analyses detected differences in communities and abundances between years, rather than between disposal and reference locations. At the scale of this research, there was no detectable impact of dredged material disposal on the epifauna communities at these two Pacific Northwest disposal sites. While the species resolution of cryptic or small organisms was found to be limited, the video sled technique had sufficient power to detect potential differences in most epifaunal species densities with a BACI statistical design. We found the video sleds were an effective tool to assess potential impacts of dredged material disposal on epifauna.

## Introduction

Under natural conditions, sediment discharging from rivers nourishes ocean beaches. However, in highly managed river systems, dredging of this material from shipping channels is usually required to maintain safe ship passage, and the sediment is commonly deposited far from littoral zones where it is needed (USACE 2012). Disposal of dredged material has thus become a global infrastructure issue requiring adaptive management solutions (Van Dolah et al. 1984; Bolam et al. 2006). One solution targets offsetting beach erosion in areas where lack of sediment supply and sea level rise coincide (Kaminsky et al. 2010), and beneficial use projects are being pursued to use dredged material to mitigate for sand loss in the littoral zone (Bolam and Whomersley 2005).

At the mouth of the Columbia River (MCR) in the Pacific Northwest of the USA, more than 3,000,000 m^3^ of material are annually dredged by the US Army Corps of Engineers (USACE) and placed at designated ocean disposal sites (Gailani et al. 2003). Historically, most dredged material has been deposited offshore where it becomes unavailable for replenishing beaches. A new program identified a method for “thin-layer” deposition of sediment at nearshore disposal sites (Wilber et al. 2007), where it can be entrained in littoral circulation and retard ongoing erosion (Oregon Solutions et al. 2011). Note this differs from direct placement on beaches. However, these nearshore soft-bottom habitats are an understudied system and questions remained about the impacts of physical forces and burial of the benthos. Notably, these nearshore areas are important habitat for economically important organisms including the Dungeness crab (*Cancer magister*), the most valuable single-species fishery in Oregon. Concerns from local fisherman and resource managers about the impacts of an expanding network of disposal sites on crab resources and navigation warranted further study (Oregon Solutions et al. 2011).

The impacts of dredged material disposal on benthic communities have been explored internationally (e.g., Jones 1986; Rees et al. 1992; Harvey et al. 1998; Valente et al. 1999; Smith and Rule 2001; Stronkhorst et al. 2003; Cruz-Motta and Collins 2004; Witt et al. 2004; Angonesi et al. 2006; Bolam et al. 2006; Powilleit et al. 2006; Vivan et al. 2009; Ware et al. 2010; Katsiaras et al. 2015; Pezy et al. 2017) and on the east (Leathem et al. 1973; Diaz and Boesch 1977; Van Dolah et al. 1984; Diaz 1994; Zimmerman et al. 2003) and Gulf coasts (Clarke and Miller-Way 1992; Flemer et al. 1997; Ray and Clarke 1999; Wilber et al. 2007, 2008) of the USA, with fewer published studies on the US west coast (McCauley et al. 1977; Blanchard and Feder 2003). The majority of these studies concentrate on impacts to macro-infaunal communities (but see Pezy et al. 2017 for a multi-trophic level analysis) and typically measure changes in species density and community composition including times to recovery to baseline conditions (see Wilber and Clarke 2007 for meta-analyses). None of these studies employed video sled technologies.

While dredged material disposal sites in the Pacific Northwest have been monitored for decades, relatively few published studies are available. McCauley et al. (1977) conducted a study at a Coos Bay, OR, dredge spoil site and found an initial increase in infaunal taxa richness and decrease in total abundance, with recovery to pre-disposal conditions within 7 days. While documenting a very rapid recovery, this study was in an estuarine, not coastal habitat where a high volume of dredge disposal is dumped. In contrast, in Alaska, Blanchard and Feder (2003) found that 6 months after disposal virtually all infaunal taxa present prior to dredging and disposal were rare or absent with opportunistic taxa dominant and that environmental conditions and faunal assemblages were still in flux 2.5 years later. Blanchard and Feder (2003) attribute some of the lack of recovery to the inability of fauna to migrate vertically through deposited sediments.

Even fewer studies report on the large, mobile epifauna that are likely to interact differently with the deposition of dredged material as compared to more sedentary infaunal organisms. A collection of historic US Army Corps studies have documented demersal fish and epibenthic invertebrate communities at the Mouth of the Columbia River disposal sites using extractive bottom trawl methods (Hinton 1998; Hinton and Emmett 1994; MEC Analytical Systems et al. 2003). While too few replicates have been taken in any survey to statistically assess disturbance and/or recovery, strong seasonal differences have been reported.

Factors affecting resilience and recovery can be classified into two categories: those relating to the disposal of the material and those relating to the habitat into which the material is disposed. In terms of disposal parameters, the time to recovery may depend on the depth of burial (Maurer et al. 1981, Maurer et al. 1982, Roberts et al. 1998, Schratzberger et al. 2000, Miller et al. 2002), the spatial scale of disturbance (Zajac et al. 1998, Guerra-Garcia et al. 2003), and the timing and frequency of deposition (Schratzberger et al. 2000). In terms of habitat, sandy sites recovery more quickly than muddy sites (Newell et al. 1998), and sites with relatively higher current speeds or wave action (which often are shallower and sandier) are expected to recover more quickly either because the communities experience natural disturbance more often or the dredged material is rapidly dispersed (Clarke and Miller-Way 1992; Hall 1994; Newell et al. 1998; Ray and Clarke 1999; Bolam and Rees 2003). Additionally, longer recovery rates have been found at higher latitudes (Blanchard and Feder 2003; Harvey et al. 1998) where long-lived taxa associated with the stable physical environments take longer to recover from disturbances (Newell et al. 1998) as compared to communities of opportunistic species more common in temperate and sub-tropical disposal areas (Van Dolah 1984, Clarke and Miller-Way 1992, Cruz-Motta and Collins 2004, Vivan et al. 2009). Finally, conclusions about disturbance and recovery may be dependent on the community sampled, as in the opportunistic versus long-lived taxa that vary with latitude, as well as the assemblage targeted for sampling within a region. For example, it has been observed that changes in meiofaunal community structure do not persist as long as changes to the macrofaunal community (Coull and Chandler 1992, Somerfield et al. 1995). Thus, Bolam et al. (2006) suggest that the exact benthic community response and recovery may be dependent on the site-specific details and should be evaluated at the site level for best management practices.

Towed video sleds have proven an effective monitoring technique for low topographic relief sedimentary habitats (Uzmann et al. 1977; Spencer et al. 2005; see Mallet and Pelletier 2014 for review of video tools). In a novel application of the method, we used towed video sleds to assess potential impacts of dredged sediment depositions on epibenthic communities as well as potential recovery time. We assessed fish and larger mobile epifaunal invertebrate densities at two study sites, one a long established deep water disposal area and the other a newly established shallow “beneficial use” site designed to aid beach sediment nourishment. To evaluate disposal effects, we developed a modified Before-After, Control-Impact (BACI) experimental design with which we compared epifaunal communities at impact (disposal) and control (reference) sites over the sediment deposition period (before, during, after) for 2 years. Both multi- and univariate analyses were used to evaluate the hypothesis testing. The goals of the study were to assess (1) potential impacts to the communities and (2) the effectiveness of the video technique for monitoring ocean disposal sites.

## Materials and methods

### Study sites

**Fig. 1:**
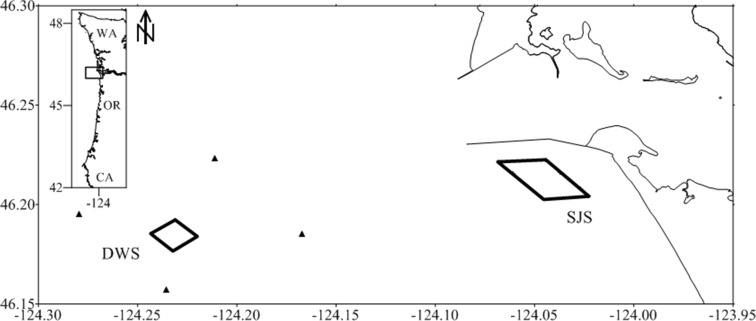
The dredged material disposal sites at the Mouth of the Columbia River (MCR). The South Jetty Site (SJS) located on the Oregon side of the mouth near the South Jetty. The Deep Water Site (DWS) is offshore with triagles denoting the boundaries of the permitted area and the square within the boundary indicates the active dumping zone throughout this study

Our study focuses on two disposal sites off the mouth of the Columbia River: the shallow water South Jetty Site (SJS) and the Deep Water Site (DWS) (Fig. 1). We surveyed both sites multiple times between August and October in both 2014 and 2015.

The SJS is located just south of the South Jetty of the Columbia River at depths ranging 12–20 m. Designated in 2012, the SJS occupies an area of 6.2 km^2^ and is considered a dispersive site where high wave energy redistributes the disposed sediment into the larger littoral cell over time (Gailani et al. 2003; USACE Portland District 2012). The SJS received 219,086 m^3^ of dredged material in 2014 and 217,573 m^3^ in 2015 (USACE Portland District and USEPA Region 10 2017). The total number of individual disposal events was similar between years, with 47 in 2014 and 50 events in 2015 (Table 1). Each disposal event was around 4200 m^3^ of non-toxic, medium- to fine-grained sand. The reference survey locations were within the boundaries of the SJS but in an area where disposal was not conducted and no effect of disposal events on the benthic community was expected (or subsequently observed).

**Table 1:** The South Jetty Site 2014–2015 video sled surveys with respect to the number of cummulative disposal events that has occured at the time of the survey

Year	Date	Disposal period	Cumulative disposal events at time of survey	Visibility	Impact transects	Control transects
2014	11 September	During	35	High	3	3
2014	1 October	After	47	High	1	3
2015	27 August	Before	0	Med-Low	3	3
2015	14 September	During	37	High	3	3
2015	25 September	During	48	High	3	3
2015	24 October	After	50	High	3	3

The DWS, designated as a disposal site in 2004, is 9.7 km offshore at 60–90 m depth and has a 17.4-km^2^ core disposal area within a 36.3-km^2^ buffer (Fig. 1). The disposal area is divided into a grid of drop zones that are rotated between years to minimize impacts. Drop Zone 14 was the designated dumping site during the study and received 1,037,770 m^3^ of dredged material in 2014 and 1,200,604 m^3^ in 2015 (USACE Portland District and USEPA Region 10 2017). Due to its depth, the DWS is a relatively low-wave energy environment and sediment does not disperse (Gailani et al. 2003; USACE Portland District 2012). Reference locations were both within the DWS where no disposal occurred and adjacent to the site at comparable depths. The total numbers of individual disposal events at DWS was 196 in 2014 and 145 in 2015 (Table 2). Like the SJS, each disposal event was around 4200 m^2^ of dredged material.

**Table 2:** The Deep Water Site 2014–2015 video sled surveys with respect to the number of cummulative disposal events that occured at the time of the survey

Year	Date	Disposal period	Cumulative disposal events at time of survey	Visibility	Impact transects	Control transects
2014	20 August	Before	0	High	3	3
2014	19 September	During	78	High	3	3
2015	19 September	During	56	High	3	3
2015	5 October	During	105	High	3	2

### Video sled design

**Fig. 2:**
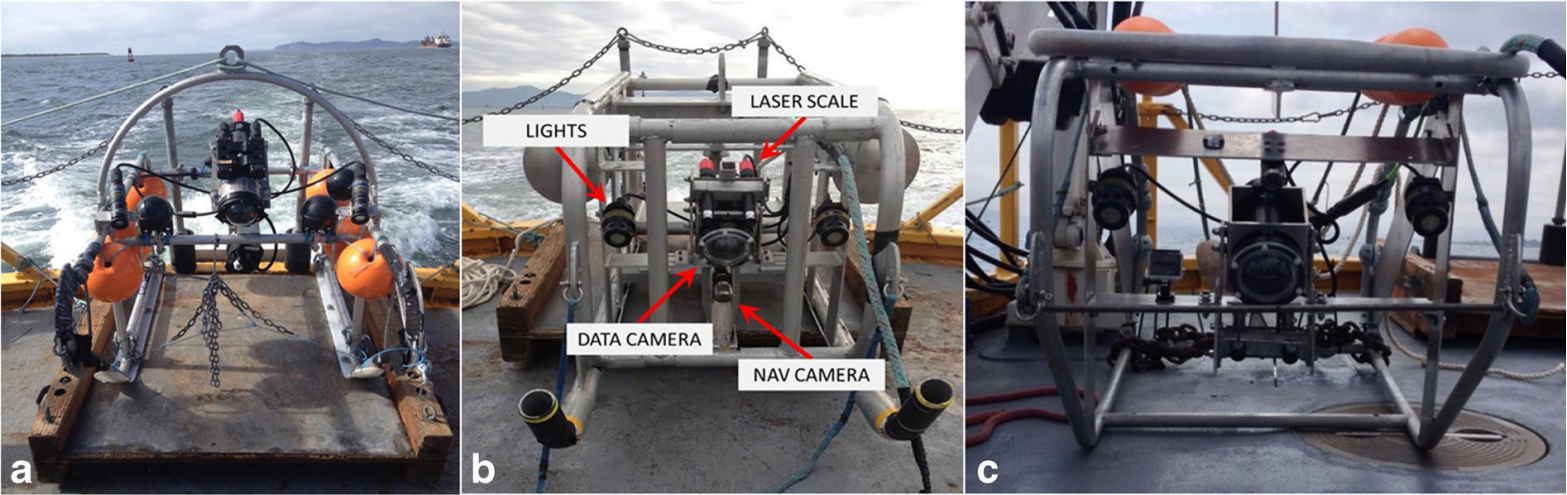
Images of the three video sleds used throughout this study. Subpanel **a** was the ODFW sled used only in the Deep Water Site in 2014, subpanel **b** is the original NOAA sled used only at South Jetty Site in 2014, and subpanel **c** is the second-generation NOAA sled used at both sites in 2015

Three video sleds were used over the 2-year study, but the instruments they carried were similar (or identical) and produced comparable results. In 2014, surveys were conducted at the DWS with the Oregon Department of Fish and Wildlife (ODFW) Marine Reserves Program’s sled (Fig. 2a) and at the SJS with the initial NOAA sled (Fig. 2b). The initial NOAA sled’s depth capacity was restricted by cable length and the ODFW sled was necessary to reach the deeper DWS depths. In 2015, a second NOAA sled (Fig. 2c) was constructed to reduce sled weight and snagging hazards and allow for deeper surveys. The video system components were consistent between the two NOAA models, and all 2015 surveys were conducted with NOAA sled-C.

The NOAA sleds had a high-resolution data camera (Canon VIXIA HF R20 camcorder) enclosed in a waterproof housing, two intensity-adjustable floodlights, and a navigation camera (Deepsea Power & Light Sealite Sphere and multi-SeaCam 2060). Additionally, two lasers (Deepsea Power & Light SeaLaser 100) were situated to provide a 10-cm measurement scale on the video imagery. Except for the data camera, which was internally powered, electrical and data transmissions to the surface were enabled through an umbilical coaxial cable. The data camera was set to a fixed focus and oriented to image a ~ 2-m^2^ area directly in front of the sled, with the laser points positioned near the center of the camera view field. The data camera recorded with progressive framing at 24 MB/s. The ODFW sled consisted of similar components that can be referenced in ODFW 2015. The ODFW sled primarily differed from the NOAA designs in camera angle and the presence of a tickler chain.

### Survey design

**Fig. 3:**
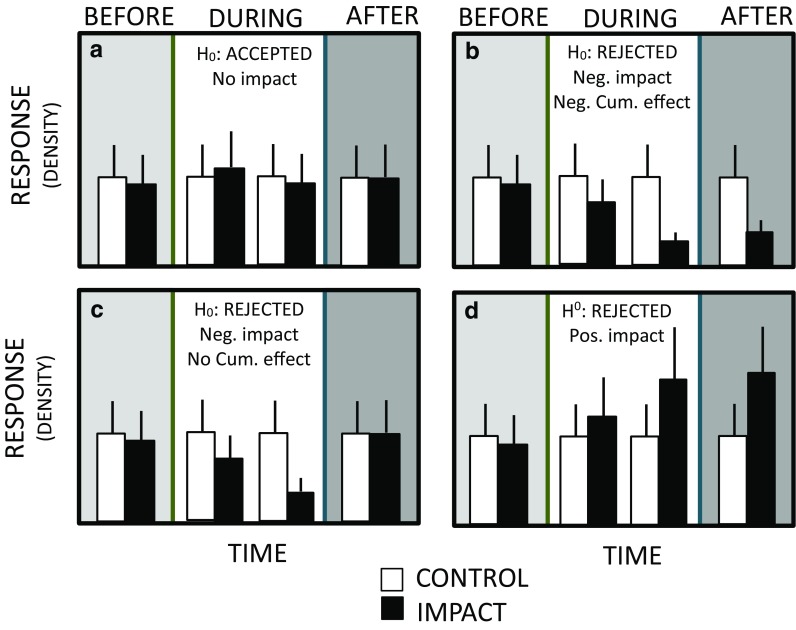
Conceptual modified BACI design outcomes

The overall experimental framework was based on a BACI design. This design calls for a comparison of two separate areas: a Control site, what we refer to as the reference site where no sediment deposition occurs, and an Impact site, the disposal site where the effects of deposition were measured. The before-after component of the design refers to the time element (before and after impact). However, for our evaluation, the “impact” was not a single event but repeated deposits of sediment that may have a cumulative effect on the benthos. Therefore, we modified the design to include a “during” phase to measure responses to sequential deposition events (Fig. 3); thus, the “after” phase may capture recovery of the benthos from impacts during the dumping season, if any. The full experimental design aimed to sample over the course of the deposition period starting before any deposition had occurred, during repeated deposits, and continuing after deposition had ceased. We used this enhanced BACI design to evaluate potential cummulative effects and possible recovery using repeated surveys of the benthos with a benthic video sled at both sites over two disposal seasons.

We developed a conceptual framework of scenarios to aid evaluating results from hypothesis testing (Fig. 3). These scenerios depict a response variable (e.g., organism density) over time in relation to the impact event (sediment deposition). In scenario A, the response variable exhibits no significant difference between before, during, or after time periods, leading to acceptance of the null hypothesis (e.g., that deposition does not affect the response variable). In scenario B, there is a decrease in the response variable during the disposal phase that continues into the after phase. This result would lead to rejection of the null hypothesis and to a conclusion that significant and cumulative negative effect had occurred. In scenario C, the response decreases during the disposal phase but rebounds after disposal ends. In this scenario, the null hypothesis would also be rejected with the conclusion that deposition had a temporary effect on the benthos. It is also possible to observe the pattern in scenario D, where the response variable increases in the during phase. This might occur if, for example, organisms were attracted to some stimulus in the deposited sediment such as organic matter entrained in the deposition material. Other patterns are also possible, for instance the control response variable may co-vary over time, due to seasonal trends in abundance.

The video sleds were used to record video transects both within disposal sites and at reference locations August to October of 2014 and 2015 (the permitted window for dredging activities in the Columbia River estuary). During each sample date, three replicate ~ 500-m transects were conducted at each disposal and reference location. Transect starting position was randomly determined, and the direction was driven by prevailing currents and wind. To ensure usable video resolution, the sled required tow speeds between 0.5 and 1.0 knots (0.25–0.50 m/s).

**Fig. 4:**
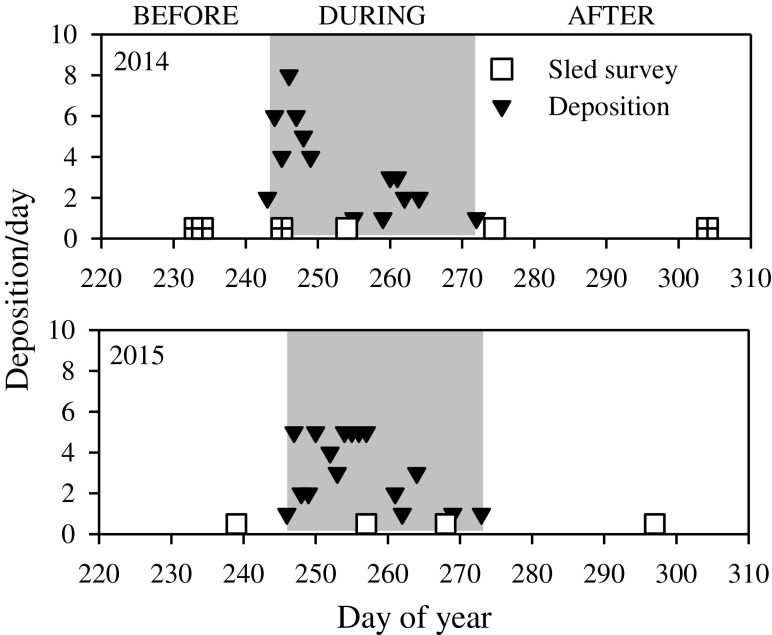
South Jetty Site dredged material disposal and video sled sampling timeline; note that the cross-hatched surveys in 2014 were not usable due to poor water clarity

At the SJS, all surveys were conducted inside the designated site boundaries, with the northern half as the reference area where no disposal activity was permitted and the southern portion as the disposal area where active disposal dumping occurred. During both years of surveys, disposal activities were limited to the month of September. At SJS, we performed sled surveys before, during, and after disposal periods in both years (Fig. 4), but poor water clarity prevented the use of three surveys in 2014, including a “before” survey, and early “during,” and a long “after” survey, reducing observations to during and shortly after disposal. In 2015, water visibility was more favorable and we were able to employ the full enhanced BACI design. Sediment deposition events were not consistent over time at SJS; most occurred in the first 7 to 10 days of dredge operations (Fig. 4).

**Fig. 5:**
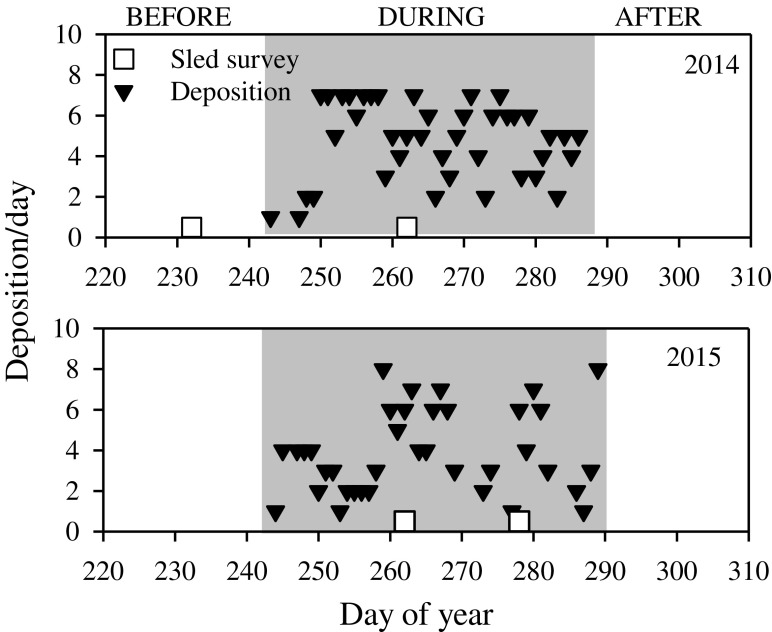
Deep Water Site dredged material disposal and video sled sampling timeline

At the DWS, the disposal area was inside the active dumping zone (the smallest rectangle in Fig. 1) and reference surveys were conducted both in the larger designated site (with no active dumping) and completely outside the site boundaries at comparable depths. During both years, disposal activities ran from late August to mid-October. The DWS consistently had good water clarity, but unfortunately, weather conditions favorable for going to sea were limited. In 2014, we conducted surveys before and during disposal—a standard BACI as we surveyed both before dumping and after 78 disposal events, while in 2015, we were only able to carry out two during disposal surveys (Fig. 5). Depositions at the DWS (the main disposal area) occurred over a longer time period each year and were more temporally consistent (Fig. 5).

### Video processing

**Fig. 6:**
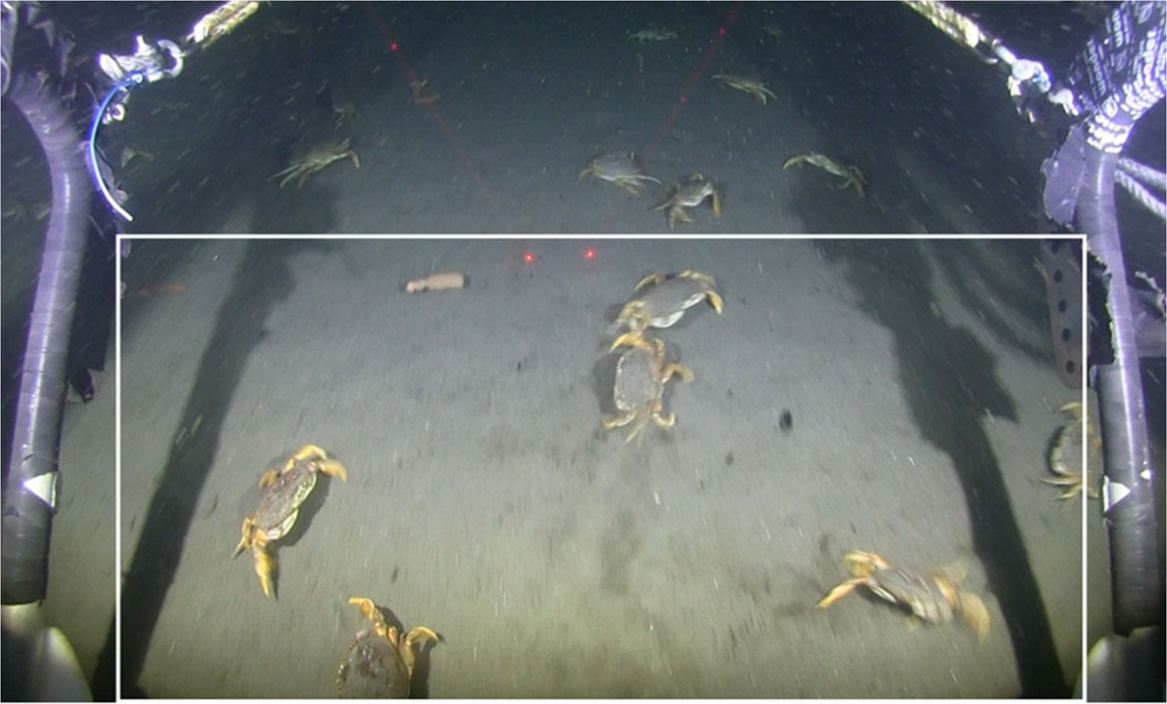
Example of the view from a video sled depicting Dungeness crab and anomalous surface distribution of unidentified spoon worms. This is the ODFW sled properly running on the seafloor and a representation of the standardized counting window (white box)

Video analysis consisted of counts of organisms, identified to the lowest possible taxon, observed in each transect. During processing, organisms were enumerated within a standardized window delineated as the area below the laser points and between the sled arms (Fig. 6, white box). Counts were only made when the sled was appropriately positioned on the bottom (laser points visibly on the seafloor) and as visibility allowed. Video processing was conducted using Windows Media Player 2010 in 2014 and with Adobe Premiere Pro CC in 2015.

Standardization of the video sled methodology necessitated adjustments due to varying sled performance. Ultimately, the area of each transect was required to calculate standardized organism densities (ind./100 m^2^). First, quantifying the width of the counting window was conducted for all three sleds. Using on-screen measurements, the ratio of actual to measured laser widths was used to calculate the actual width of countable area:

$$ {W}_{\mathrm{A}}={W}_{\mathrm{M}}\times {L}_{\mathrm{A}}/{L}_{\mathrm{M}} $$

where *W*_A_ is the actual window width (m), *W*_M_ is the screen-measured window width, and *L*_A_/*L*_M_ is the ratio of the actual laser width to the screen measured laser width.

The initial NOAA sled (2014 SJS surveys) consistently stayed on the bottom, so the window width was easily determined by a single ratio measurement (0.85 m). The other two sleds were lighter and sometimes ascended into the water column (sometimes for large portions of the transect) which altered the window-to-laser width ratio. Consequently, we calculated the ranges of widths for each sled. The ODFW sled (2014 DWS surveys) ranged almost 2 m in width depending on vertical position (1.2–3.0 m above the seabed). To address this issue and determine the best width reflected over all DWS 2014 transects, five random samples of the ratio were calculated for each transect and the mean value was used for the respective transect width. The range of the second NOAA sled (used for both sites in 2015) was narrow, with < 0.01 m difference between the closest and furthest useable positions. We used 0.57 m width (the furthest) for all calculations.

Second, transect length needed to be determined from using start and end latitude and longitude, assuming straight transect paths (generally true). This method did not necessarily reflect the usable distance covered by the sled (as some transects had segments of low or no visibility when flying off the bottom). Thus, we corrected the total distance traveled by the proportion of usable time, assuming a constant velocity (generally true) as:

$$ {A}_{\mathrm{T}}=L\times {W}_{\mathrm{A}}\times \left({T}_{\mathrm{U}}/{T}_{\mathrm{T}}\right) $$

where *A*_T_ (m^2^) is the total measurable area, *L* the distance traveled (m), *W*_A_ the window width (m), and proportion of usable time (*T*_U_/*T*_T_) is the proportion of usable time to total time (min). Organism density was then calculated using the corresponding transect area (*A*_T_) and then standardized to ind./100 m^2^.

Prior to statistical analysis, data were sorted by removing non-targeted habitat (a rocky segment on a DWS reference survey), pelagic species (e.g., baitfish), and unidentified species (e.g., unidentified invertebrates).

### Data analysis

Separately for each survey site (SJS and DWS), cluster dendrograms (“group average” method) and Multidimensional Scaling plots (MDS) were constructed using a Bray-Curtis similarity matrix on log-transformed density data. In creating the cluster dendrogram, a Similarity Profile Analysis (SIMPROF) test was run to determine whether statistically distinct groups existed within the similarity matrix. Similarity percentage (SIMPER) analysis was run to determine which species contributed the most to similarity in the groups determined by the SIMPROF procedure. Two-way Analyses of Similarities (ANOSIM) were conducted to determine if there were statistically significant differences in community compositions. The ANOSIM output includes a global *R* value; values closer to 1 indicate greater differences between levels of a factor and values closer to 0 indicate more similarity between levels. The initial goal was to conduct an ANOSIM on factors of disposal period (before, during, after) and location (disposal and reference); however, as noted above, the usable observations resulted in unbalanced data for the disposal period. Due to this and interest in exploring differences between the two sampling years, we proceeded with factors of year (2014 and 2015) and location (disposal and reference). All multivariate analyses were conducted in the statistical program PRIMER 6 (Clarke 1993).

Univariate approaches were used to explore individual taxon responses to disposal impacts. Due to the unbalanced survey, we used one-way ANOVAs to test the factor Location (disposal and reference) on taxa densities only during the disposal period for each site separately (focus on the white panel of Fig. 3). At both the SJS and the DWS, we had three “during” disposal surveys, each with three replicates, for a total of nine observations each in the disposal and reference locations. For each of these sites, taxa occurring in at least four observations during the disposal periods were tested. In 2015 at SJS, we achieved the full enhanced BACI survey design and were able to test for differences in mean densities of prominent organisms between disposal and reference locations before, during, and after the disposal season (Table 1). In 2014 at DWS, we achieved a traditional BACI where we had one survey before dumping began and one survey after 78 disposal events during the disposal season (Table 2). Two-way analysis of variance (ANOVA) tests were conducted with the response variable of taxa density and explanatory variables of location (disposal and reference) and date of survey in 2015 for SJS and 2014 for DWS; Tukey HSD post hoc tests were used to determine which levels of factors were significantly different. Density data were ln transformed when necessary to better meet the assumptions of equal variance. All analyses were conducted in R statistical software (R Core Team 2013) at the *p =* 0.05 significance level.

## Results

### Video processing

Identification of mega-invertebrates to species was more successful than identification of fish to species. Fish identification was a challenge at the video image resolution, especially for small individuals (< 5 cm). For flatfish (Pleuronectiformes), positive confirmation of species was extremely difficult and all were combined in a single group. Six species of flatfish are reported in the DWS area from 2014 trawl surveys, including Pacific sanddab (*Citharichthys sordidus*), rex sole (*Glyptocephalus zachirus*), slender sole (*Lyopsetta exilis*), and Dover sole (*Solea solea*) (Marine Taxonomic Services 2015). The sculpin (Cottoidae) category for the DWS may contain poachers (Agonidae) due to difficulty discerning small fish. Sculpin species may include slim sculpin (*Radulinus asprellus*) and staghorn sculpin (*Leptocottus armatus*) and poachers may include pricklebreast poacher (*Stellerina xyosterna*) and pygmy poacher (*Odontopyxis trispinosa*) (Marine Taxonomic Services 2015). Because of this uncertainty in fish identification, we did not pursue analysis of species metrics such as richness or diversity.

Of the common invertebrates, hermit crabs were also difficult to identify to species and were grouped as Paguridae. Note that the video resolution did allow for the positive identification of buried Dungeness crab, which were especially prevalent at the DWS.

### South Jetty Site

A total of 4878 organisms and nine taxa were observed on SJS surveys (Table 3). Dominant species/taxa groups were Dungeness crab, hermit crabs, whelks, and flatfish. Infrequent observations included octopus (*Octopus rubescens*)—observed only in 2014—and sand dollar, sand lance (Ammodytidae), and skate (*Raja* spp.), which were unique to 2015. Sculpins were occasionally observed in both years. The dense sand dollar (*Dendraster excentricus*) “bank” accounted for more than three-quarters of the total counts. Organisms/groups occurring frequently enough for univariate statistical analysis were Dungeness crab, hermit crab, whelks, sand dollars, and flatfish.

**Table 3:** South Jetty Site mean taxa densities (/100 m^2^)

Common name	Scientific name	September 11, 2014 (during)		October 1, 2014 (after)		August 27, 2015 (before)		September 14, 2015 (during)		September 25, 2015 (during)		October 24, 2015 (after)	
		Disposal	Reference	Disposal^a^	Reference	Disposal	Reference	Disposal	Reference	Disposal	Reference	Disposal	Reference
Dungeness crab	*Cancer magister*	1.84	1.70	0.71	0.71	2.01	2.18	1.19	1.93	0.62	2.90	0.12	0.12
Hermit crab	*Paguridea* spp.	6.57	2.78	3.09	3.15	2.31	0.63	8.01	8.13	3.49	8.39	14.90	22.44
Sand dollar	*Dendraster excentricus*	–	–	–	–	–	–	451.22	0.12	0.12	13.97	0.12	0.12
Octopus	*Octopus rubescens*	–	–	0.24	0.08	–	–	–	–	–	–	–	–
Whelk	*Nucella* spp.	0.53	0.15	1.19	0.39	0.12	–	0.57	0.54	–	0.38	0.71	0.78
Flatfish	Pleuronectiformes	1.53	1.07	1.19	0.70	1.00	1.97	0.26	0.12	0.84	0.62	–	0.14
Sand lance	Ammodytidae	–	–	–	–	–	–	0.14	–	0.12	–	0.23	–
Sculpin	Cottoidei	–	0.08	–	0.47	–	–	–	0.12	–	–	0.12	–
Skate	*Raja* spp*.*	–	–	–	–	–	–	–	–	0.12	–	–	–
Total taxa	Total taxa	4	5	5	6	4	3	6	6	6	5	6	5

Most columns represent the mean densities of three replicate transects

^a^Densities for the only usable transect

**Fig. 7:**
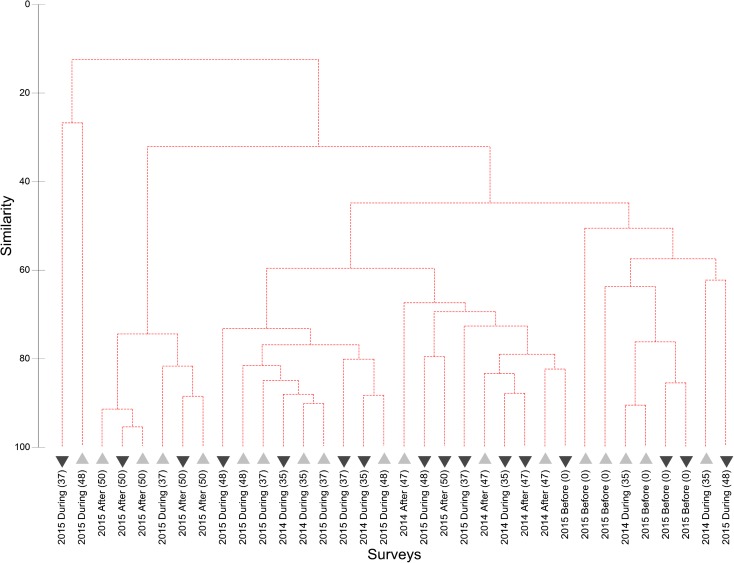
Cluster dendrogram of SJS communities observed on video sled transects. The dotted lines indicate statistically similar groups based on the SIMPROF test. Lighter upward triangles are reference locations and darker down triangles are disposal locations. Labels indicate the year and location of the survey, with the parenthesis indicating the number of cumulative disposal events at the time of the sled survey

**Fig. 8:**
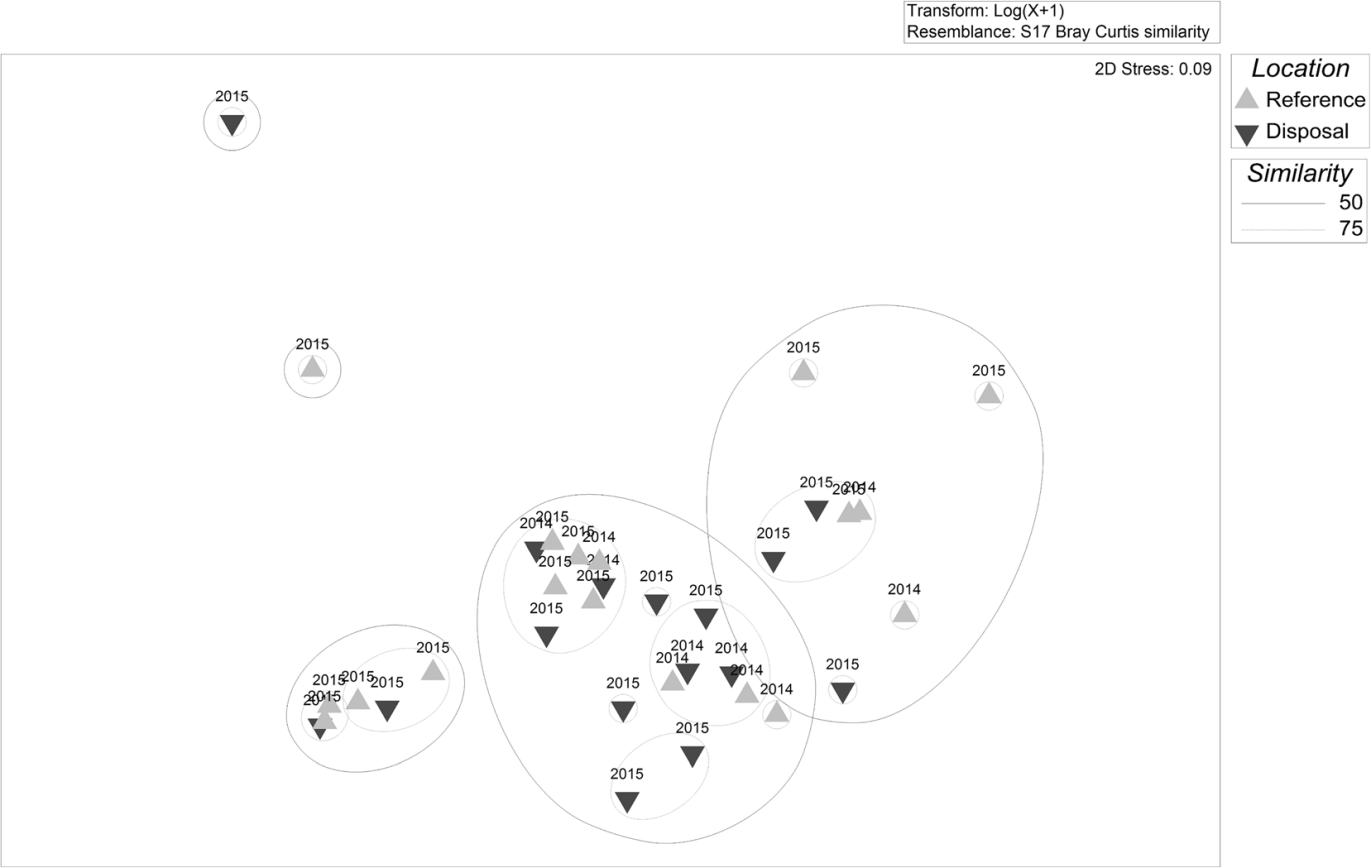
MDS plot of SJS communities observed on video sled transects. The distance between points reflects the similarity of the communities. The transect labels indicate the year of the survey

The cluster analysis with SIMPROF test resulted in no significant differences between any SJS transects, with no structure apparently related to disposal or reference areas, years, or timing of observation relative to disposal (Fig. 7). The two transects that were the outliers from the main cluster both contained high densities of sand dollars and consisted of one transect from the disposal area and one from the reference area; thus, the aggregation of sand dollars did not seem to be related to disposal. The MDS plot also depicts the disposal and reference surveys mostly overlapping (Fig. 8); even 50% similar (solid lines) and 75% similarity (dotted lines) groups contained a mix of reference and disposal surveys. Here the two outliers are as not tightly clustered since the reference transect had relatively lower sand dollar densities as compared to the one disposal transect, making it more similar to the rest of the stations. The SIMPER analysis revealed that the average similarity between transects was 45.90%, with hermit crabs contributing the most to total similarity (67.45%), followed by Dungeness crab (17.26%). An ANOSIM with factors of Year (*R* = − 0.038, *p* value = 0.594) and Location (*R* = 0.051, *p* value = 0.129) resulted in *R* values both near zero, which would indicate high similarity among years and across locations. The non-significant *p* value (> 0.05) requires accepting the null hypothesis that there is no evidence that species compositions differ between Years or Locations.

**Fig. 9:**
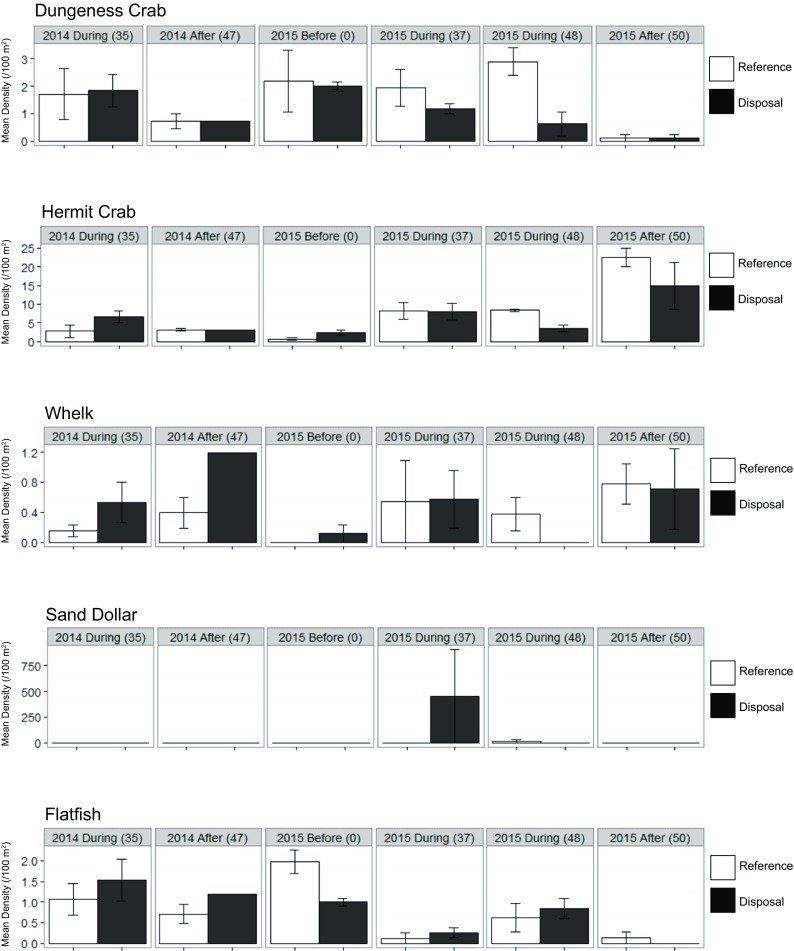
Mean densities (/100 m^**2**^) of frequently occurring taxa of the South Jetty Site. Standard error bars (±SE) are displayed and the number in the parenthesis indicates the number of cumulative disposal events experienced by the disposal location

At SJS, the one-way ANOVAs for the “during” disposal period across both years found no evidence of statistically significant differences in densities of Dungeness crabs (*p =* 0.069), hermit crabs (*p =* 0.804), sand dollars (*p =* 0.721), whelks (*p =* 0.968), or flatfish (*p =* 0.388) between reference and disposal locations during the disposal period, which was our main hypothesis (Table 4). The nearly significant effect of location on Dungeness crab was likely because numbers in 2014 were equivalent between locations, while in 2015, there were less crabs in the disposal location in the latter part of the dumping season (Fig. 9).

**Table 4:** One-way ANOVA results of all *during* disposal surveys at SJS for the factor of Location

Taxa	Factor	*Df*	Sum Sq	Mean Sq	*F* value	*P* value	ES	Power
Dungeness crab	Location	1	4.178	4.178	3.811	0.069	1.130	0.994
	Residuals	16	17.543	1.096				
Hermit crab	Location	1	0.744	0.744	0.064	0.804	3.322	1.000
	Residuals	16	186.841	11.678				
Sand dollar^a^	Location	1	0.479	0.479	0.132	0.721	318.619	1.000
	Residuals	16	57.854	3.616				
Whelks	Location	1	0.0005	0.00045	0.0017	0.968	0.501	0.515
	Residuals	16	4.2686	0.267				
Flatfish^a^	Location	1	0.107	0.107	0.787	0.388	0.677	0.770
	Residuals	16	2.182	0.136				

The effect size (ES) is the standard deviation of taxa densities observed across locations and used in the post hoc power analyses

^a^ln transformed densities used in the analysis

Using two-way ANOVAs on only the 2015 data (when we had the enhanced BACI survey design), Dungeness crab densities varied significantly by date (*p =* 0.005) while location (*p =* 0.054) and the interaction (*p =* 0.068) were both borderline significant (Table 5). Crab densities were significantly lower in the last survey as compared to the three previous with no differences detected between other pairs of survey dates. The interaction was significant because both the reference and the disposal locations after dumping were different from the reference location during the second dumping period. All other pairs of interactions were insignificant. We also detected significant differences in mean density of hermit crab (Paguridae) by date (*p* < 0.001) and the interaction of location and date (*p =* 0.035) in 2015. Among dates, hermit crab densities during disposal (across locations) were similar to each other, and significant differences were detected between all other pairs of dates, with hermit crabs increasing through the survey period in 2015. For the interaction of location and date, similar densities at both locations before dumping and lower densities in the disposal during the disposal season would indicate an effect of dumping. However, Tukey HSD indicated that hermit crab densities did not differ significantly between locations during either the first (*p =* 1.0) or second (*p =* 0.396) “during” survey. Neither sand dollar nor whelk densities varied significantly by location, date, or the interaction. Flatfish densities varied significantly (*p* < 0.001) between sampling dates with only the first “during” and the “after” samples being statistically similar; however, average densities were low overall (< 2 fish/100 m^2^), so differences among sampling dates likely are not ecologically meaningful.

**Table 5:** Two-way ANOVA results of the 2015 surveys at SJS for factors of Location and Date when we achieved the enhanced BACI survey design (bold type indicates statistically significant *p*-values (< 0.05))

Taxa	Factor	*Df*	Sum Sq	Mean Sq	*F* value	*P* value	ES	Power
Dungeness crab^a^	Location	1	3.8311	3.8311	4.528	0.054	1.249	0.999
	Date	3	13.679	4.560	5.388	**0.005**		
	Location/date	3	4.857	1.619	1.913	0.068		
	Residuals	16	13.539	0.846				
Hermit crab^a^	Location	1	0.157	0.157	0.867	0.366	7.838	1.000
	Date	3	13.093	4.364	24.047	**< 0.001**		
	Location/date	3	2.000	0.667	3.674	**0.035**		
	Residuals	16	2.904	0.182				
Sand dollar^a^	Location	1	0.360	.0360	0.134	0.720	276.059	1.000
	Date	3	6.266	2.089	0.775	0.525		
	Location/date	3	10.176	3.392	1.259	0.322		
	Residuals	16	43.125	2.695				
Whelks	Location	1	0.035	0.0345	0.107	0.748	0.561	0.866
	Date	3	1.811	0.604	1.865	0.176		
	Location/date	3	0.207	0.069	0.213	0.886		
	Residuals	16	5.179	0.323				
Flatfish^a^	Location	1	0.317	0.317	1.736	0.498	0.688	0.965
	Date	3	1.993	0.664	3.638	**< 0.001**		
	Location/date	3	0.699	0.233	1.277	0.142		
	Residuals	16	2.922	0.183				

The effect size (ES) is the standard deviation of taxa densities observed across locations and used in the post hoc power analyses

^a^ln transformed densities were used in the analysis

Since neither ANOVA analyses resulted in significant *p* values for the factor of Location (our main concern), post hoc power analyses were conducted to ascertain our ability to detect an effect size greater than the observed variation (standard deviation). The results indicate we had sufficient power for detecting an effect size greater than or equal to the variation we detected across locations for all species at SJS except whelks (Tables 4 and 5). In summary, we found marginally significant effects of disposal on Dungeness crab 2015; however, these findings are in contrast to 2014 when during the disposal season densities were higher (if not significantly so) in the disposal area as compared to the reference area.

### Deep Water Site

A total of 6013 organisms and 18 taxa were observed on all DWS surveys (Table 6). Almost a quarter of the observations were spoon worms (Echiura) from three 2014 transects. Spoon worms were not only unique to 2014 but also to the disposal site location. The orange sea pen (*Ptilosarcus gurneyi*), giant pink sea star (*Pisaster brevispinus*), ribbon worm (Nemertea), sea whip (Pennatulacea), sunflower star (*Pycnopodia helianthoides*), and skate (*Raja stellulata*) also were unique to 2014 (all with very low counts). Hermit crabs (Paguridae) were the only taxon unique to 2015 (also low counts). The taxa occurring frequently enough for univariate analysis were Dungeness crab, white plumose anemone, nudibranch, sand star, flatfish, eelpout, lingcod, and sculpin.

**Table 6:** Deep Water Site mean densities (/100 m^**2**^)

Common name	Scientific name	August 20, 2014 (before)		September 19, 2014 (during)		September 19, 2015 (during)		October 5, 2015 (during)	
		Disposal	Reference	Disposal	Reference	Disposal	Reference	Disposal	Reference^a^
Dungeness crab	*Cancer magister*	1.06	1.00	17.67	1.96	0.42	3.65	1.60	7.75
Hermit crab	*Paguridea* spp*.*	–	–	–	–	0.19	–	0.36	–
Giant plumose anemone	*Metridium giganteum*	0.19	0.15	–	0.65	0.58	0.67	–	0.36
Sea anemone	Actiniaria	–	0.04	–	0.11	–	–	–	0.18
Nudibranch	Heterobranchia	0.03	–	–	–	0.96	1.60	–	13.55
Orange sea pen	*Ptilosarcus gurneyi*	–	–	–	0.04	–	–	–	–
Sea whip	Pennatulacea	0.05	0.08	–	0.08	–	–	–	–
Giant pink star	*Pisaster brevispinus*	–	–	–	0.04	–	–	–	–
Sand star	*Luidia foliolata*	1.07	0.94	0.80	0.85	3.68	1.76	3.49	2.49
Sunflower star	*Pycnopodia helianthoides*	0.03	–	0.04	–	–	–	–	–
Weathervane scallop	*Patinopecten caurinus*	0.11	–	–	–	0.19	0.12	0.13	0
Spoon worm	Echiura	–	–	58.00	–	–	–	–	–
Ribbon worm	*Nemeretea*	–	0.04	–	–	–	–	–	–
Flatfish	Pleuronectiformes	25.15	17.60	11.01	15.27	28.42	25.92	45.26	51.88
Sculpin	Cottoidei	4.83	3.74	0.52	0.91	1.34	1.16	0.52	0
Eelpout	Zoarcidae	9.73	4.83	0.07	2.48	–	0.41	–	1.48
Lingcod	*Ophidon elongatus*	–	0.25	0.07	0.11	0.19	–	0.13	0.47
Starry skate	*Raja stellulata*	–	0.04	–	–	–	–	–	–
Total taxa		10	11	8	11	9	8	7	8

Most columns represent the mean densities of three replicate transects

^a^Two replicates

**Fig. 10:**
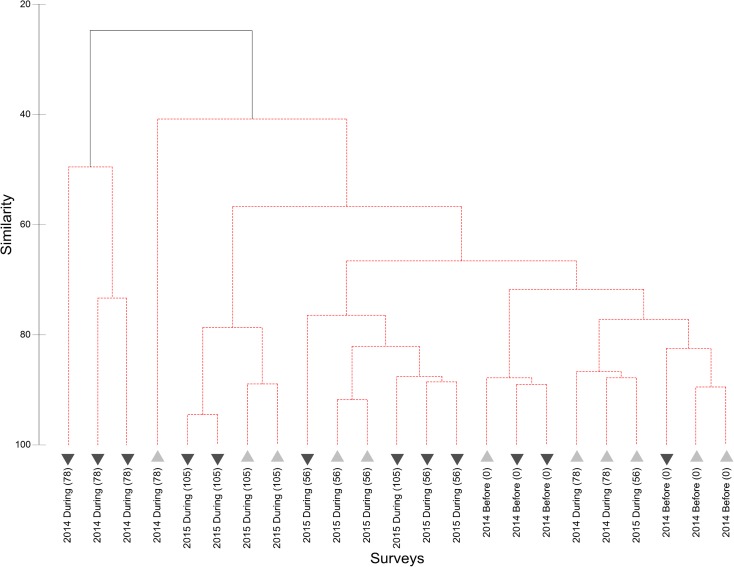
Cluster dendrogram of DWS communities observed on video sled transects. The solid line indicates statistically different groups based on the SIMPROF procedure. Lighter upward triangles are reference locations and darker down triangles are disposal locations. Labels indicate the year and location of the survey, with the parenthesis indicating the number of cumulative disposal events at the time of the sled survey

**Fig. 11:**
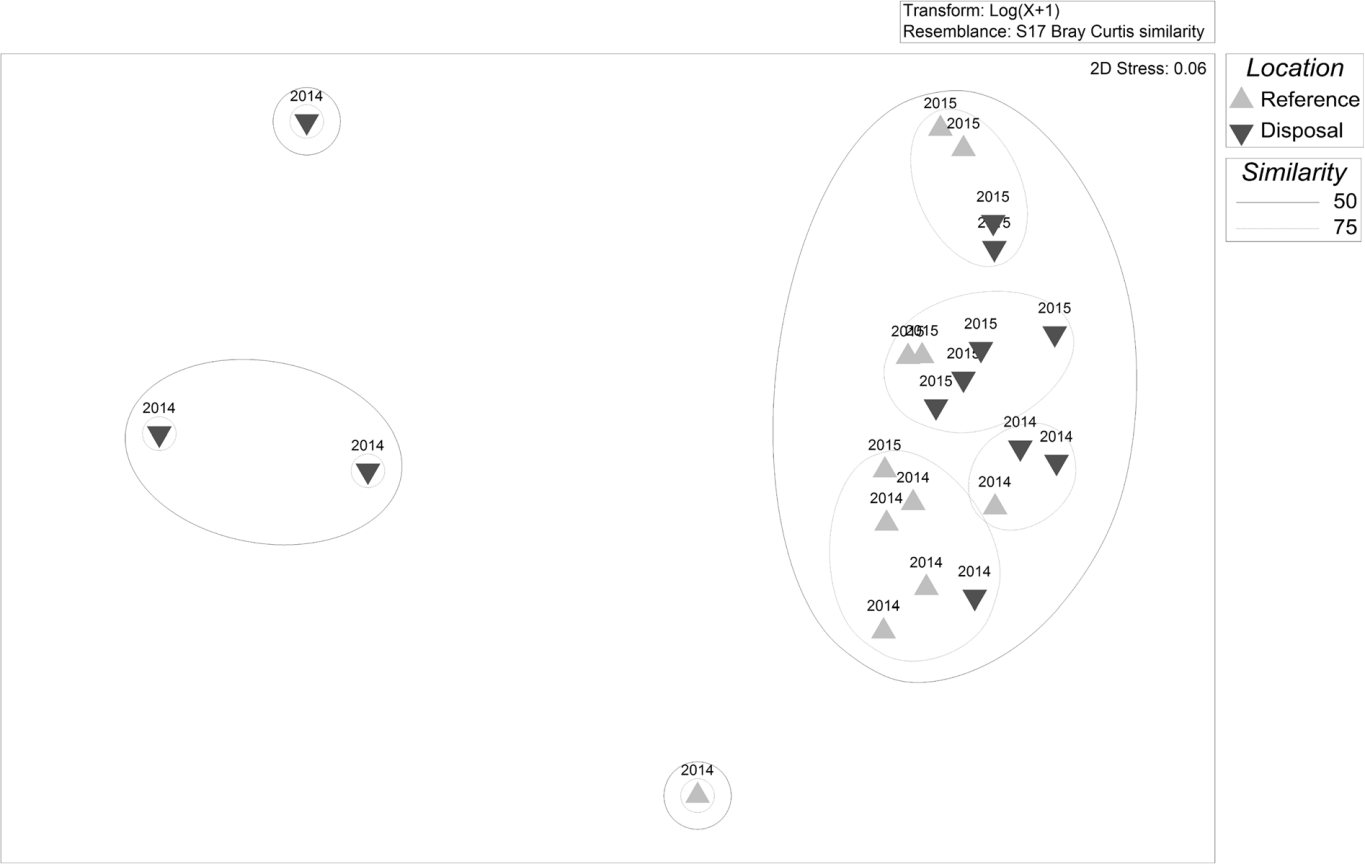
MDS plot of DWS communities observed on video sled transects. The distance between points reflects the similarity of the communities. The transect labels indicate year of the survey

The cluster analysis and SIMPROF test resulted in two significantly distinct groups at the DWS (Fig. 10). The division separates the three September 2014 disposal location transects with high spoon worm densities from all other surveys. The average *dissimilarity* between the two groups was 75.28%, with the spoon worm contributing over half (54.64%), followed by flatfish (21.57%), Dungeness crab (12.83%), and eelpouts (3.70%). Average spoon worm and Dungeness crab abundance were higher in the outlying group, while flatfish and eelpout (Zoarcidae) abundances were higher in the main group. To check the degree to which the anomalous spoon worms were affecting the results, we re-ran the cluster analysis without spoon worms. There was almost no change to the structure of the dendrogram because of the high densities of Dungeness crabs found spatially overlapping with the spoon worms (just two stations swapped positions), and the difference between the three stations on the left and the rest of the dendrogram was no longer significant (plot not shown). The MDS plots of the full dataset revealed that the three high spoon worm and Dungeness crab transects were the greatest distance from the other points but also relatively distant from each other (Fig. 11). All other 2014 transects except one were in a tight group, which are separated from the 2015 samples in both the dendrogram and MDS plot. At a 50% similarity level, almost all of the other disposal and reference surveys grouped together. Even at 75% similarity, as at the SJS, the smaller groups were still composed of both disposal and reference surveys. Together, these analyses indicate greater variability in the observed communities between years than between disposal versus reference areas. These interpretations are confirmed by the ANOSIM, which revealed a significant (*p =* 0.001) *R* value of 0.442 for the factor year, indicating that communities within each year were more similar than between years. For Location, the *R* value was just significant (*R* = 0.164, *p =* 0.050); however, the value was quite low indicating that most samples were similar across locations (disposal versus reference), again suggesting little impact to epifauna in the disposal zone.

**Fig. 12:**
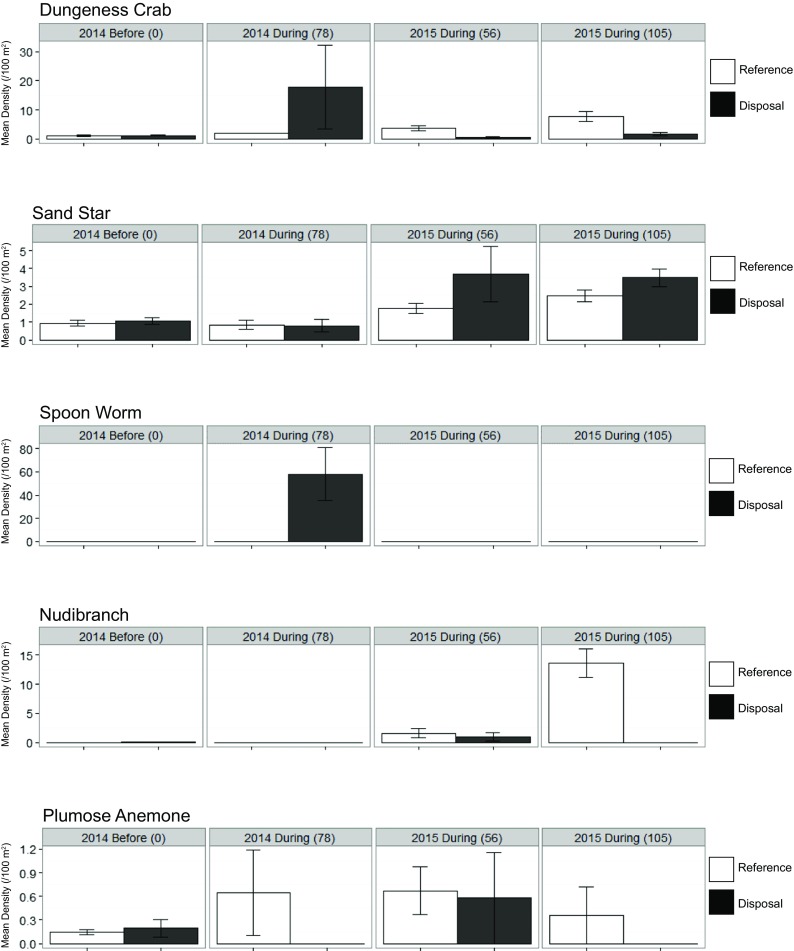
Mean densities (/100m^**2**^) of frequently occurring invertebrate taxa of the Deep Water Site. Standard error bars (±SE) are displayed and the number in the parenthesis indicates the number of cumulative disposal events experienced by the disposal location

**Fig. 13:**
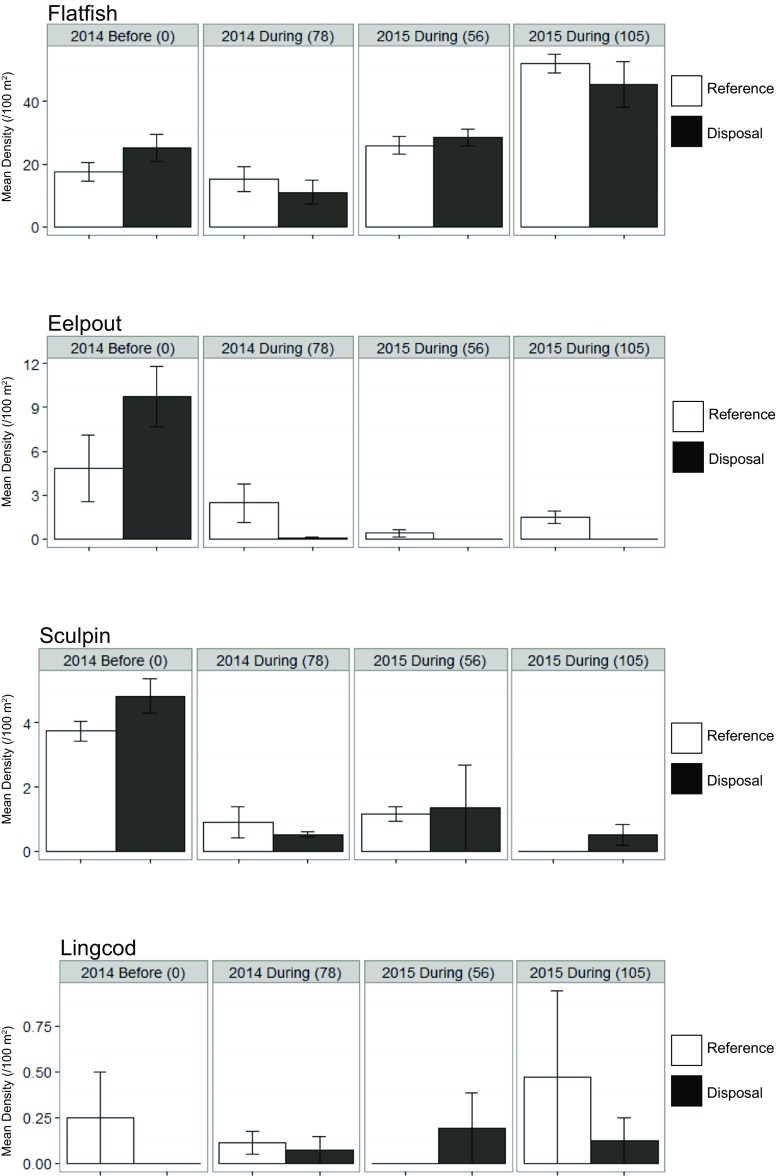
Mean densities (/100m^2^) of frequently occurring fish taxa of the Deep Water Site. Standard error bars (±SE) are displayed and the number in the parenthesis indicates the number of cumulative disposal events experienced by the disposal location

Dungeness crab densities were higher at the disposal site in the 2014 surveys, concurrent with high spoon worm densities (Fig. 12). In 2015, Dungeness densities were lower in the disposal locations; however, the average density increased in both locations in the second sampling after more disposal. Combining the “during” observations from both years into a one-way ANOVA on Location indicated no significant effect of disposal on Dungeness crab (*p =* 0.392), plumose anemone (*p =* 0.198), nudibranch (*p =* 0.094), or sand stars (*p =* 0.366) (Table 7 and Fig. 12). Among fish, flatfish (*p =* 0.982), lingcod, (*p =* 0.830), and sculpin (*p =* 0.819) densities were statistically indistinguishable between locations when combining “during” observations across both years. Only eelpouts had statistically significant different densities between locations during disposal (*p =* 0.003), being almost entirely absent from the disposal locations (Table 7 and Fig. 13). Thus, with respect to our question comparing reference and disposal locations, we only detected evidence of statistically significant differences in the densities for one taxon.

**Table 7:** One-way ANOVA results of all *during* disposal surveys at DWS for the factor of Location (bold type indicates statistically significant *p*-values (< 0.05))

Taxa	Factor	*Df*	Sum Sq	Mean Sq	*F* value	*P* value	ES	Power
Dungeness crab^a^	Location	1	0.648	0.648	0.775	0.392	10.857	1.00
	Residuals	15	12.536	0.836				
Plumose anemone^a^	Location	1	0.233	0.233	2.405	0.136	0.533	0.177
	Residuals	15	2.032	0.098				
Nudibranch	Location	1	2.781	2.781	3.979	0.065	4.502	1.00
	Residuals	15	10.4839	0.699				
Sand star^a^	Location	1	0.222	0.222	0.869	0.366	1.60	0.868
	Residuals	15	3.833	0.256				
Flatfish	Location	1	0.148	0.148	< 10^−3^	0.982	15.806	1.00
	Residuals	15	3997.3	266.486				
Eelpout^a^	Location	1	2.147	2.147	12.588	**0.003**	1.281	0.693
	Residuals	15	2.558	0.171				
Lingcod	Location	1	0.019	0.019	0.467	0.502	0.238	0.081
	Residuals	15	0.860	0.041				
Sculpin^a^	Location	1	0.012	0.012	0.054	0.819	1.011	0.495
	Residuals	15	3.395	0.226				

The effect size (ES) is the standard deviation of taxa densities observed across locations and used in the post hoc power analyses

^a^ln transformed densities were used in the analysis

Using two-way ANOVAs on only the 2014 data (when we had the standard BACI survey design; Table 8), no significant effects were detected for the invertebrate taxa, although our power to detect differences for plumose anemone and sand star was low (Table 8). Visually, there do not appear to be negative effects on sand star, while the plumose anemone may be negatively affected by disposal (Fig. 12). If power is calculated using the difference of means/variance, the power to detect a change in densities of plumose anemone by location was 0.9995.

**Table 8:** Two-way ANOVA results of the 2014 surveys at DWS for factors of Location and Date when we achieved the standard BACI survey design (bold type indicates statistically significant *p*-values (< 0.05))

Taxa	Factor	*Df*	Sum Sq	Mean Sq	*F* value	*P* value	ES	Power
Dungeness crab^a^	Location	1	0.949	0.949	1.476	0.259	12.974	1.00
	Date	1	2.592	2.592	4.031	0.080		
	Location/date	1	0.785	0.785	1.220	0.302		
	Residuals	8	5.145	0.643				
Plumose anemone^a^	Location	1	0.102	0.102	1.346	0.279	0.477	0.567
	Date	1	0.006	0.006	0.086	0.778		
	Location/date	1	0.139	0.139	1.839	0.212		
	Residuals	8	0.606	0.076				
Sand star	Location	1	0.006	0.006	0.031	0.866	0.380	0.397
	Date	1	0.092	0.092	0.499	0.500		
	Location/date	1	0.022	0.022	0.122	0.736		
	Residuals	8	1.471	0.184				
Flatfish^a^	Location	1	0.0001	0.0001	0.001	0.981	7.773	1.00
	Date	1	0.788	0.788	4.526	0.0661		
	Location/date	1	0.318	0.318	1.828	0.213		
	Residuals	8	1.393	0.174				
Eelpout^a^	Location	1	0.054	0.054	0.180	0.682	4.477	1.00
	Date	1	6.078	6.078	20.319	**0.002**		
	Location/date	1	2.177	2.177	7.277	**0.027**		
	Residuals	8	2.393	0.299				
Lingcod	Location	1	0.062	0.062	1.150	0.315	0.218	0.164
	Date	1	0.003	0.003	0.053	0.823		
	Location/date	1	0.033	0.033	0.620	0.454		
	Residuals	8	0.429	0.054				
Sculpin	Location	1	0.374	0.374	0.780	0.403	2.000	1.00
	Date	1	38.165	38.165	79.636	**< 0.001**		
	Location/date	1	1.629	1.629	3.400	0.102		
	Residuals	8	3.834	0.479				

The effect size (ES) is the standard deviation of taxa densities observed across locations and used in the post hoc power analyses

^a^ln transformed densities were used in the analysis

Among fish, eelpouts again appeared to be affected by disposal with significant responses both Date (*p =* 0.002) and the interaction of Location and Date (*p =* 0.027). Sculpins varied significantly by Date in 2014 (*p* < 0.001), with lower densities at both disposal and reference locations during the disposal season as compared to before. However, neither Location (*p =* 0.403) nor the interaction (*p =* 0.102) was significant. No significant effects were detected for flatfish or lingcod. Using standard deviation, the power to detect differences for lingcod was low; however, if power is calculated using the difference of means/variance, the power to detect a change in densities of lingcod by location was 1, and the variance does not appear to be related to the factors we tested (Fig. 13). Thus, it appears that the only taxon significantly affected by disposal at the DWS was eelpouts.

## Discussion

### Benthic community responses

We collected epibenthic species density data from benthic video transects at two ocean disposal sites with reference locations using a BACI experimental design. At the South Jetty Site, we achieved an enhanced design that included a “during” phase so that we could assess both the potential acute impacts of dredged material disposal and, if detected, determine if communities recovered in the “after” phase, similar to the approach of Katsiaras et al. (2015). While we did not achieve our planned experimental design at both sites in both years due to challenging ocean conditions, our surveys had sufficient power to detect potential differences in most species densities between disposal and reference locations. Multivariate analyses assessed communities as a whole and indicated greater temporal than spatial (reference/disposal) differences in benthic taxa densities, with no consistent differences in benthic assemblages between disposal and reference areas were detected at either site in 2014 or 2015.

Analyses of nine individual taxa across both sites indicated possible effects on Dungeness crabs (at the South Jetty Site only) and significant effects on eelpout (at the Deep Water Site) (Table 9). During disposal periods, many of the frequently occurring taxa responded differently at disposal sites and between years. Together, these results indicate that most effects of sediment disposal on epibenthic community composition or individual species abundances were less than seasonal or interannual differences.

**Table 9:** Summary of the effect of disposal on primary epibenthic species abundance based on BACI design hypothesis testing

Result	Species	Conclusion
No effect	Whelks	Highly variable
	Sand dollar	Highly variable
	Sand star	Not significantly higher at disposal locations during dumping in 2015
	Nudibranch	Highly variable
	Flatfish	Significant temporal variability present at disposal and reference locations
	Sculpin	Declined over time both years at both disposal and reference locations
	Lingcod	Highly variable
Potential effect	Dungeness crab	Statistical significance for effect of location marginal, recovery from any potential effects rapid
	Hermit crab	Statistical significance marginal, recovery from potential effects rapid
	Plumose anemone	No statistical support but graphs are suggestive
	Eelpout	Essentially absent from disposal areas during disposal. Highly significant because of high 2014 “before” abundance

Our lack of detectable responses in epifaunal assemblages and densities of most individual taxa surveyed is likely due to factors related to disposal parameters, the receiving habitat, and the communities assessed. The sediment being disposed is similar in grain size to the offshore habitats: the MCR navigation channel sediment averaged 98.45% sand when sampled in 2008 (USACE 2008) while offshore locations consisted of > 99% sand when sampled in 2000 (McLaren and Hill 2000). Further, the high quality sediment had no contaminant detected at or near screening levels (USACE 2012). In addition to the sediment characteristics, at the South Jetty Site, thin-layer disposal techniques are employed, resulting in less accumulation in a concentrated area and reducing burial depths (Wilber et al. 2007).

The shallower, high-energy SJS was characterized by relatively low abundances of very few epifaunal taxa and showed no differences in community composition between disposal and reference areas. The thin-layer disposal technique used there coupled with a naturally dispersive setting likely results in minimal effects from burial, as physically dynamic habitats have been documented to recover from disturbance relatively quickly compared to deeper and more stable systems (Clarke and Miller-Way 1992; Hall 1994; Newell et al. 1998; Ray and Clarke 1999; Bolam and Rees 2003). The statistical results supported scenario A for flatfish, sand dollars, and whelks and potentially scenario C for Dungeness and hermit crabs (Fig. 3). Our findings are similar to Bolam et al. (2011) where dominant species were very similar regardless of proximity to disposal area at a dispersive, coastal disposal site in the southwest UK. As in our multivariate analysis, the Bolam et al. (2011) study revealed few differences in community structure between stations inside and those outside the disposal site, which was attributed to the dispersive nature of the site. At our nearshore site, the epibenthos are larger mobile organisms (fishes and crab) or are capable of shallow burial in shifting sediments (hermit crabs and gastropods). Thus, the community is expected to be adapted to periods of sediment resuspension due to wave surge, and although the sediment disposal plume is a unique stressor (Roegner and Fields 2015), impact effects appear ephemeral. The potential acute effects on Dungeness crabs in 2015 lacked persistence after the disposal season and were not present in 2014.

While the DWS is considered to be a non-dispersive site, meaning sediment is expected to remain within the site over time due to the relatively low wave energy environment (Gailani et al. 2003, USACE Portland District 2012), few effects were detected here either. Video observations of the sediment plume at the seafloor revealed dispersal of the material as it descended 70 m through the water column (authors’ unpublished data). As at SJS and in the findings by Bolam et al. (2011), the multivariate analysis did not detect differences in the communities related to disposal. In terms of individual species, no invertebrate taxa had significant responses to disposal, with the possible exception of plumose anemones for which we had low power to detect differences. As the anemones are one of the only sessile species observed, and the plots seem to indicate a negative effect, further research on this species is warranted.

Among fishes, flatfish densities at the DWS were similar between disposal and reference locations throughout our surveys and were more abundant than at SJS. Lingcod and sculpin similarly seemed unaffected by dredged material disposal at the DWS. Although our power to detect responses in these species was low, the plots indicate equal or higher densities of sculpin in disposal areas in most cases. Eelpout, however, did appear to respond negatively to the dredged material disposal, demonstrating a response similar to the white panel of scenarios B and C in our conceptual outcomes (Fig. 3). Without surveys after disposal at the DWS, we cannot determine if there is a cumulative effect or if eelpouts recovered at the DWS disposal location after cessation of dumping. The viviparous eelpout (*Zoarces viviparous*) has been proposed as a key indicator organism in the Baltic and North Sea for monitoring anthropogenic effects (OSPAR, 2007; HELCOM, 2008; Hedman et al., 2011). Contrary to our observed potential negative effects of dredged material disposal on eelpouts, Langhamer et al. (2018) found no negative effects of an offshore wind farm on eelpouts. Thus, this taxon may be more affected by changes to sediment conditions than the introduction of hard structure to sedimentary habitat.

Both sand dollars at SJS and spoon worms at the DWS were spatio-temporally ephemeral but of high abundance. Sand dollars were observed in “banks” several meters wide but many unknown meters long in which the sand dollars were densely packed and oriented vertically (i.e., on their edges). This orientation reportedly aids in particle capture during suspension feeding (O’Neill 1977). When sampling these linear features with the video sled, abundance counts depend on the angle the sled intersects the bank, with higher abundances as the angle deviates from perpendicular. We observed these features at only two transects—one disposal and one reference—and at present, we do not have enough data on the spatio-temporal distribution of these echinoderms to evaluate disposal impact effects.

**Fig. 14:**
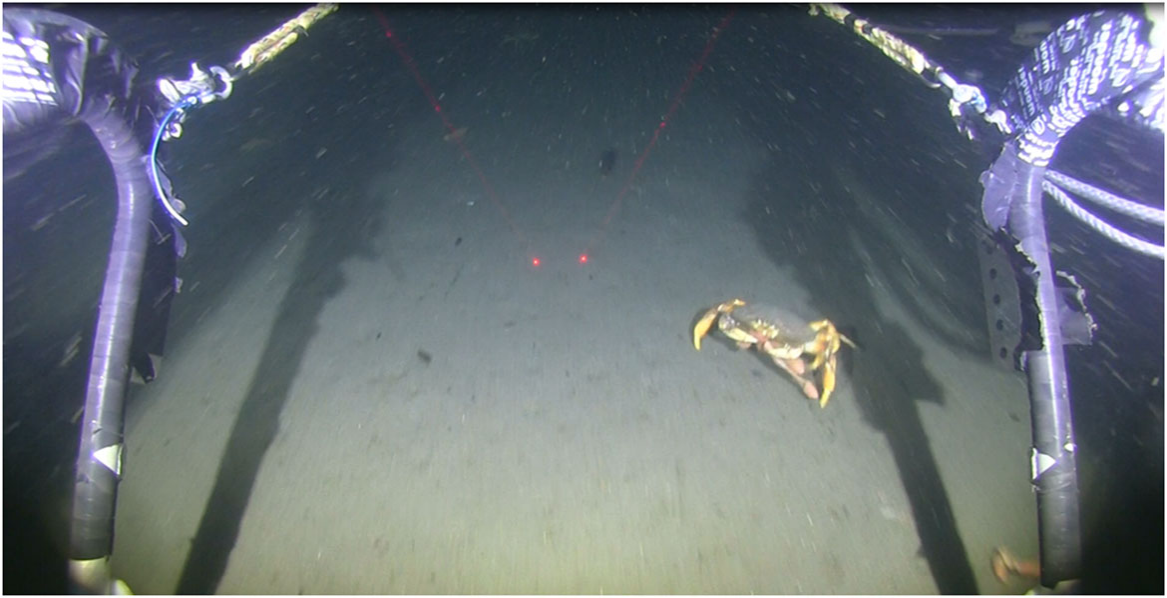
An example of Dungeness crab grasping unidentified spoon worm at Deep Water Site in 2014 during the anomalous observation of spoon worms present in high densities on the surface of the seafloor

An extremely anomalous spoon worm distribution was also observed on one occasion at the impact treatment at the DWS in 2014—anomalous because the echiurans were widely scattered on the surface of the seafloor rather than buried within it. This made them easy prey for predators and likely explains the very high Dungeness crab densities we measured concurrently as crabs were observed carrying worms in their chelae (visible in Fig. 14). The cause of the disinterment is unclear: low dissolved oxygen is known to affect biota on the Oregon shelf, or warm water anomalies during 2014 could have caused a mass exodus from the substrate. However, the occurrence was only observed in the impact treatments and it is unknown if the echiuran presence was restricted to the deposition zone (e.g., had a positive association to the deposits). These observations have not to our knowledge been previously reported.

Bottom trawl surveys were conducted by the US Environmental Protection Agency at the DWS in 2014 within the same disposal location as our video surveys but during different time periods (Marine Taxonomic Services 2015). Most organisms collected in the trawls were identified to species, and, in agreement with our study, the authors found greater differences in the fish and invertebrate communities between the July and October sampling than between the disposal and non-disposal areas within the same time period. Historical MCR monitoring work has also attributed variability in infaunal community compositions near the DWS region to seasonal changes and fluctuating sediment stability at the mouth of the river (Hinton and Emmett 1994) as sediment structure has been found to vary due to river discharge, primary productivity, and storms (Richardson et al. 1977). At the DWS where we were able to compare methods, both benthic trawl and video surveys demonstrated temporal (seasonal or interannual) differences in communities superseded spatial variability related to dredged material placement. With such high seasonal and interannual variability in the MCR system, increased survey effort across the modified BACI design would increase the robustness of the observed patterns.

### Use of video sleds at disposal sites

Water visibility was the primary limitation to utility of the video sled, as it is with all video systems. Even when weather and sea state allowed for deployment of the sled, turbidity at the seafloor was variable, especially in the nearshore zone. In 2014 at the South Jetty Site, there were four surveys of low visibility that did not allow for census data to be extracted. In 2015, conditions improved and all four deployments were usable. In contrast, visibility at the Deep Water Site in both years had clear conditions across all surveys. This suggests using video tools in deeper sites might be more dependable for water clarity compared to shallow sites in our area of study. Phytoplankton blooms contributed significantly to periods of reduced visibility, and predicting or measuring nearshore water quality characteristics may aid in predicting favorable sled conditions in the future.

The video sleds proved to be useful for detecting variation in epifaunal abundances, with the caveat that identification to species was limited for the smaller or cryptic organisms, as found by Sheehan et al. (2010). Identifying fish to species was mostly unachievable, since many species, particularly those found in sedimentary habitats, are discernable by only small-scale characteristics requiring close discernment (e.g., Pacific sanddab versus speckled sanddab). However, note that at SJS, the overall densities of flatfish were also very low, so species-level identification would likely not have increased the information value for impact comparisons. One way to address the species identification limitation in the future would be to incorporate a high-resolution still camera system into the sled design to increase image resolution. We conclude that for future community surveys of shallow sites, video sled surveys are a preferred method where non-extractive procedures are warranted.

While the video sled does lack the species resolution of a trawl, it was superior for ease of deployment/retrieval, had a rapid turn-around, and could sample for long periods (limited mainly by battery consumption of our data camera, which could have been engineered to be powered through the coaxial cable). The sled allowed for identification of buried and fleeing Dungeness crab, which would not be captured by traditional trawl methods, and beam trawl sampling has been found to underestimate population assessments of Dungeness crab when compared to video sled or SCUBA diver surveys (Spencer et al. 2005). Similarly, when comparing trawl to video surveys for thornyhead rockfish, Lauth et al. (2004) found the video sled recorded mean densities three to five times higher than the trawl data. Importantly, the video sled allows for unique observations of spatially associated organisms (e.g., Dungeness crab and spoon worms, sand dollar banks), which would be homogenized in extractive trawl samples. Finally, the processing time of the videos must be considered. Processing rates varied depending on the density of organisms on the transect, but for the 2015 data, there was a 3:1 ratio of processing time to transect run time. This compares to the trawl’s average ratio of 10:1 processing to transect time (Marine Taxonomic Services, Ltd., personal communication). These are clear benefits for video sleds over trawls.

Future surveys should use a single survey tool (one sled) to avoid discrepancies between tools. We accounted for these differences by calculating a standardized metric allowing for comparisons between sled surveys. Improvements include a time code generator/GPS tracking synced to the video camera to provide more exact transect distance calculations and more fine-scale data on taxa distributions at the disposal sites (every observation would have a spatial component) (Knight et al. 2014).

### Conclusions

We found the video sleds were an effective tool to survey epifaunal species of interest to assess potential impacts of dredged material disposal without further impacting the organisms by extraction. We had sufficient power to detect potential differences in most of the epifaunal species surveyed but found few taxa differed significantly in their observed densities in patterns attributable to disposal. Overall, temporal differences exceeded sediment disposal effects. Thus, we conclude that epifaunal fish and invertebrate communities are not significantly negatively affected by the methods of dredged material disposal utilized at the mouth of the Columbia River, where clean sand is deposited offshore in dispersive and/or deep areas.
